# Amelioration of Particulate Matter-Induced Oxidative Stress by a Bioactive *Hizikia fusiformis* Extract: A Functional Biomaterial for Cosmeceutical Applications

**DOI:** 10.3390/md23030135

**Published:** 2025-03-20

**Authors:** Jeong Won Ahn, Hyun Soo Kim, So Hui Kim, Hye Soo Yang, Kongara Damodar, Yeong-Min Yoo, Jin Tae Hong, Seong Soo Joo

**Affiliations:** 1 College of Life Science, Gangneung-Wonju National University, Gangneung 25457, Gangwon-do, Republic of Korea; 0000@gwnu.ac.kr (J.W.A.); gustn4609@gwnu.ac.kr (H.S.K.); kongaradamu@gwnu.ac.kr (K.D.); 2 East Coast Life Sciences Institute, Gangneung-Wonju National University, Gangneung 25457, Gangwon-do, Republic of Korea; yooym@gwnu.ac.kr; 3 R&D Center, Happy L&B Co., Ltd., Icheon 17405, Gyeonggi-do, Republic of Korea; shkim@happylnb.com (S.H.K.); didgptn11@happylnb.com (H.S.Y.); 4 College of Pharmacy, Chungbuk National University, Chungju 28644, Chungbuk-do, Republic of Korea; jinthong@chungbuk.ac.kr; 5 Huscion MAJIC R&D Center, Seongnam 13488, Gyeonggi-do, Republic of Korea; 6 Environmental Research Institute, Kangwon National University, Chuncheon-si 24341, Gangwon-do, Republic of Korea

**Keywords:** *Hizikia fusiformis*, urban particulate matter, oxidative stress, anti-inflammatory, fucoidan

## Abstract

Air pollution-related skin damage has heightened the demand for natural protective agents. *Hizikia fusiformis*, a brown seaweed rich in fucoidan and bioactive fatty acids (α-linolenic acid, eicosatetraenoic acid, and palmitic acid), possesses antioxidant and anti-inflammatory properties. This study investigated the protective effects of *H. fusiformis* ethanol extract (HFE) against particulate matter (PM)-induced oxidative stress, inflammation, and apoptosis in human keratinocytes. Antioxidant activity was assessed using DPPH and hydroxyl radical scavenging assays, while PM-induced cytotoxicity, ROS generation, inflammatory markers, and apoptotic pathways were evaluated using the WST-8 assay, DCFH2-DA, qPCR, western blotting, and Hoechst staining. HFE significantly reduced ROS levels, enhanced antioxidant enzyme activity, and mitigated PM-induced cytotoxicity. These effects were mediated by fucoidan and fatty acids, which modulated inflammatory pathways (NF-κB and MAPK), stabilized membranes, and inhibited apoptosis (Bcl-2, Bax, and caspase-3). Collectively, these findings highlight HFE’s potential as a natural anti-pollution skincare ingredient, supporting further in vivo studies and formulation development.

## 1. Introduction

Urban particulate matter (PM) is a complex mixture of solid and liquid particles and a primary source of air pollution, especially in densely populated regions, such as China, Korea, and Japan [1,2]. Increasing levels of fine dust in these areas have amplified public health concerns, particularly regarding its effect on skin health [3,4,5]. Research has increasingly shown that PM exposure is a critical risk factor for skin diseases, such as atopic dermatitis and premature aging, mainly by inducing oxidative stress and inflammatory responses [6].

As the body’s primary barrier against environmental hazards, the skin relies on complex signaling pathways to maintain cellular homeostasis [2,7]. However, PM particles (≤2.5 μm in diameter) can penetrate these defenses and disrupt this balance, leading to cellular damage, characterized by oxidative stress and inflammation [6,7,8,9]. Such exposure can trigger apoptosis, alter cellular biochemistry and morphology, and ultimately compromise skin barrier function [8,10]. The increasing prevalence of PM-induced skin conditions highlights the need for effective protective strategies. Natural compounds, including polyphenols and flavonoids, have emerged as promising low-toxicity options to mitigate these effects [8,9,11,12].

Recent studies have focused on identifying natural agents that can counteract the harmful effects of PM on skin health [8]. *Hizikia fusiformis* (Harvey) Okamura, a brown seaweed species commonly found along the rocky coastlines of East Asia, has attracted considerable attention owing to its lucrative bioactive properties [13]. Rich in bioactive compounds, including polysaccharides, fucoidan, fucosterol, and phenols, *H. fusiformis* demonstrates notable anti-tumor, immunomodulatory, anti-inflammatory, and antioxidant activities [13,14,15,16,17,18]. These characteristics make them particularly valuable in protecting the skin from PM-induced damage [13]. Research has shown that extracts from *H. fusiformis* modulate inflammatory responses, inhibit the activation of nuclear factor κ-light-chain-enhancer of activated B cells (NF-κB), and reduce the generation of reactive oxygen species (ROS), thereby helping to preserve cellular integrity and function [18,19].

Our previous research revealed that *H. fusiformis* extracts contain novel bioactive compounds with potent anti-atopic dermatitis properties [14,20]. Fucoidan, a sulfated polysaccharide found in brown seaweeds, exhibits notable antioxidant and anti-inflammatory effects, as well as biosorption capabilities, making it particularly effective against oxidative stress induced by PM [9,21,22,23]. These properties suggest that *H. fusiformis* could be an effective natural remedy for mitigating inflammatory and oxidative stress caused by environmental pollutants. However, the specific mechanisms through which *H. fusiformis* protects against PM-induced skin damage are poorly understood.

The present study aimed to elucidate the mechanism by which *H. fusiformis* extract mitigates PM-induced cellular damage in human keratinocytes by examining its regulatory effects on key inflammatory markers and apoptotic pathways. These findings may contribute to the development of natural formulations that protect against air pollution-related skin damage.

## 2. Results and Discussion

### 2.1. Antioxidant Activities

**Figure 1:**
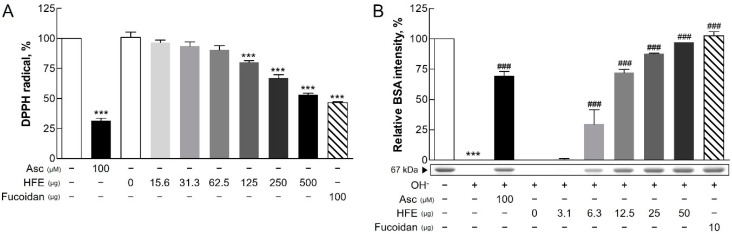
Antioxidant activity of HFE. (**A**) DPPH radical scavenging activity (%) of HFE was assessed at doses ranging from 15.6 to 500 µg, showing a maximum activity of approximately 60% at 500 µg. Ascorbic acid (Asc, 100 μM) exhibited the highest radical scavenging activity, significantly greater than HFE at all tested doses. (**B**) Protection against hydroxyl radical-induced BSA protein degradation (%) was evaluated at doses ranging from 3.1 to 50 µg of HFE, with optimal protection reaching approximately over 90% at 50 µg. Fucoidan (10 µg) showed protective effects similar to the highest HFE dose. Ascorbic acid (100 μM) and fucoidan served as positive and reference controls, respectively. Data represent the mean ± SD from three independent experiments. *** *p* < 0.001 vs. control; ### *p* < 0.001 vs. the hydroxyl radical (OH-) group. HFE, *H. fusiformis* ethanol extract; DPPH, 2,2-diphenyl-1-picrylhydrazyl; BSA, bovine serum albumin.

The antioxidant activity of HFE was evaluated using two complementary assays. In the DPPH radical scavenging assay, HFE exhibited dose-dependent antioxidant activity, with significant effects observed at 125 μg. While ascorbate (control antioxidant) exhibited higher activity in the DPPH assay, HFE showed comparable activity at 500 μg. Additionally, similar antioxidant effects were observed with fucoidan at 100 μg (Figure 1A). A hydroxyl radical scavenging assay using BSA as a protein substrate demonstrated that HFE significantly inhibited hydroxyl radical-induced protein degradation in a dose-dependent manner (Figure 1B). These results collectively indicated the potent antioxidant capacity of HFE, consistent with previous study findings [15].

The protective effects of HFE against PM-induced damage are primarily attributed to its high fucoidan content. Fucoidan operates through multiple mechanisms, including reducing ROS levels and suppressing key inflammatory pathways, such as mitogen-activated protein kinase (MAPK) and NF-κB signaling [24,25]. This suppression leads to the reduced production of pro-inflammatory cytokines and alleviates oxidative stress and inflammation [26]. The significant free radical scavenging activity observed in both antioxidant assays confirmed the antioxidant properties of fucoidan, which complemented the overall protective effects of HFE. These findings suggested that *H. fusiformis*, through its fucoidan content, could serve as an effective therapeutic agent for PM-induced oxidative stress-related skin conditions [10].

### 2.2. Effects of HFE on Cell Viability and Oxidative Stress Induced by PM

**Figure 2:**
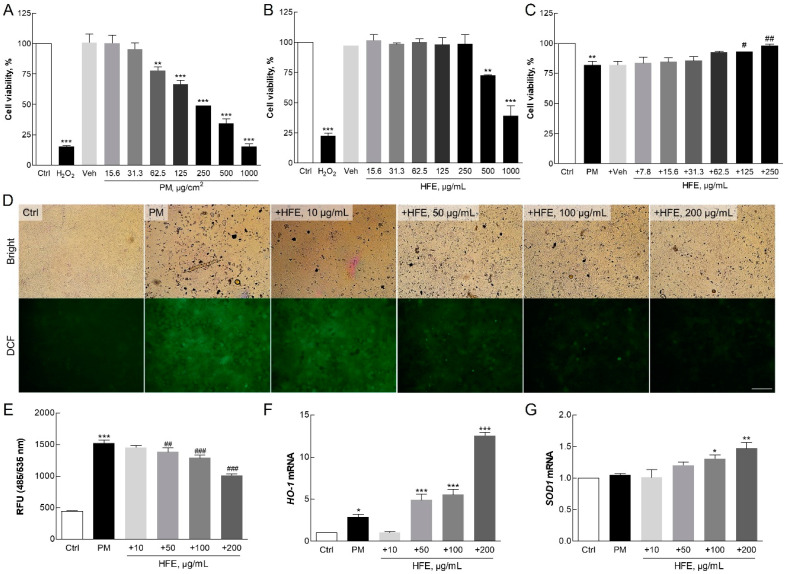
Effect of HFE on PM-induced oxidative stress in HaCaT cells. (**A**–**C**) Cell viability was measured using the WST-8 assay after 24 h of exposure to PM (0–100 µg/cm^2^), HFE (0–500 µg/mL), or HFE with 50 µg/cm^2^ PM. (**D**–**E**) Intracellular ROS levels were measured using DCFH2-DA fluorescence in cells pre-treated with HFE (1 h) followed by PM exposure (50 µg/cm^2^, 30 min). Scale bar = 100 µm. (**F**–**G**) mRNA levels of antioxidant enzymes HO-1 and SOD1 were analyzed using qPCR after HFE pre-treatment (1 h) and PM exposure (24 h). Data represent the mean ± SD (*n* = 3). * *p* < 0.05, ** *p* < 0.01, *** *p* < 0.001 vs. control; # *p* < 0.05, ## *p* < 0.01, ### *p* < 0.001 vs. PM group. HFE, *H. fusiformis* ethanol extract; PM, particulate matter; ROS, reactive oxygen species.

Cytotoxicity in HaCaT cells was assessed using the WST-8 assay. PM showed significant cytotoxic effects at concentrations above 62.5 µg/mL, while HFE exhibited cytotoxicity only at concentrations exceeding 500 µg/mL (Figure 2A,B). Notably, HFE mitigated PM-induced cytotoxicity in a dose-dependent manner, with 250 µg/mL of HFE maintaining approximately 100% cell viability (Figure 2C). This cytoprotective effect likely stems from the bioactive compounds in HFE that enhance cell survival via ROS modulation [19].

The ROS assay indicated a significant reduction in ROS generation in HaCaT cells pre-treated with HFE prior to PM exposure compared with that in the control group, which exhibited significantly higher ROS levels (Figure 2D,E). The extract demonstrated a dose-dependent inhibition of ROS production, with the greatest reduction, approximately 40%, observed at 200 µg/mL. This reduction in ROS levels is crucial for skin health because excessive ROS can lead to oxidative damage, thereby accelerating skin aging and promoting inflammation [27].

**Figure 3:**
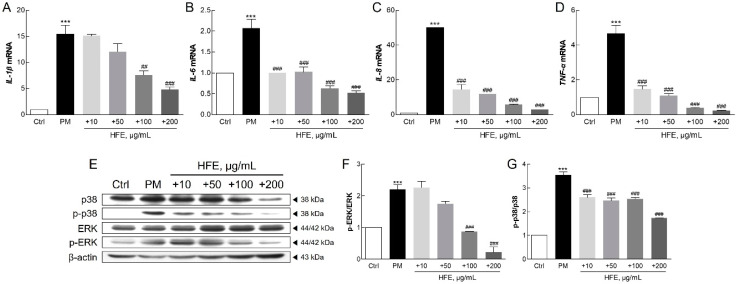
Effect of HFE on the PM-induced inflammatory response in HaCaT cells. (**A**–**D**) mRNA levels of pro-inflammatory cytokines (IL-1β, IL-6, IL-8, and TNF-α) were analyzed after HFE pre-treatment (1 h) and PM exposure (50 µg/cm^2^, 24 h). (**E**–**G**) Phosphorylation levels of ERK and p38 MAPK were assessed with Western blot after HFE pre-treatment (1 h) and PM exposure (1 h). Data represent the mean ± SD (*n* = 3). *** *p* < 0.001 vs. control; ## *p* < 0.01, ### *p* < 0.001 vs. PM group. HFE, *H. fusiformis* ethanol extract; PM, particulate matter; IL, interleukin; TNF-α, tumor necrosis factor α; ERK, extracellular signal-related kinase; MAPK, mitogen-activated protein kinase.

Furthermore, HFE increased the levels of the antioxidant enzymes heme oxygenase 1 (HO-1) and superoxide dismutase 1 (SOD1) in a dose-dependent manner (Figure 3F,G). Several intracellular antioxidant enzymes, including HO-1 and SOD1, play crucial roles in regulating the balance between oxidant and antioxidant species in mammalian cells [28]. HO-1 is an inducible enzyme responsible for heme breakdown and is a critical antioxidant. It is highly upregulated in response to pro-oxidant and pro-inflammatory stimuli, thereby providing protection against oxidative damage [29]. The expression of HO-1 is largely regulated by the redox-sensitive transcription factor, nuclear factor erythroid 2-related factor 2 (NRF2), which binds to the antioxidant response element (ARE) in the promoter regions of many antioxidant genes [30]. SOD is the only enzyme that can eliminate superoxide radicals (O_2_^−^) from mammalian cells and serves as a key defense mechanism against oxidative stress [28]. Consistent with previous study findings on fucoidan-containing extracts, the fucoidan present in HFE is thought to play a pivotal role in enhancing antioxidant responses [9].

Our results demonstrate that HFE effectively protects cells against PM-induced oxidative stress through two mechanisms: direct inhibition of ROS production and enhancement of cellular antioxidant defense systems. These findings suggested that HFE effectively maintains skin integrity by counteracting PM-induced oxidative damage.

### 2.3. Effects of HFE on PM-Induced Inflammatory Response via the MAPK Signaling Pathway

qPCR analysis revealed that PM significantly upregulated key inflammatory markers in HaCaT cells, including interleukin 1β (IL-1β), IL-6, IL-8, and tumor necrosis factor α (TNF-α), while HFE treatment effectively suppressed their expression (Figure 3A–D). Furthermore, HFE reduced the phosphorylation of MAPK proteins, particularly p38 and ERK1/2, indicating its inhibitory effect on this signaling pathway (Figure 3E–G).

PM can bind to toll-like receptor 5 (TLR5) on keratinocytes, initiating a signaling cascade that activates NF-κB. This activation regulates the expression of target genes involved in the inflammatory response [31]. Furthermore, PM-induced NF-κB activation leads to the transcription of pro-inflammatory cytokines, such as IL-1, IL-6, IL-8, and TNF-α [31,32]. Previous studies have reported that these genes play critical roles in acute inflammation and the pathogenesis of inflammatory skin diseases [33,34,35,36]. PM exposure also activates the MAPK pathway, which is characterized by the phosphorylation of ERK, JNK, and p38 in keratinocytes [36]. Activation of the p38 MAPK pathway is essential for inflammatory cytokine production and signaling [37]. In keratinocytes, PM-induced IL-1 secretion mediates dermal collagen degradation via a p38 kinase-dependent pathway [38]. The extracellular signal-related kinase (ERK) signaling pathway further regulates the induction of chemokines, cytokines, and adhesion molecules that contribute to, and sustain, the inflammatory response [39]. Additionally, recent research has underscored that the MAPK signaling pathway plays a crucial role in PM-induced apoptosis, further illustrating the breadth of cellular mechanisms affected by PM [40].

Our findings demonstrated that HFE effectively suppresses PM-induced inflammation through two mechanisms: downregulation of pro-inflammatory gene expression and inhibition of MAPK activation. These results established that *H. fusiformis* possesses potent anti-inflammatory properties, mediated through the modulation of both the NF-κB and MAPK pathways. This mechanism aligns with previous studies on natural seaweed extracts and their effectiveness in mitigating pollutant-induced inflammation [36].

### 2.4. Protective Effects of HFE Against PM-Induced Apoptosis

**Figure 4:**
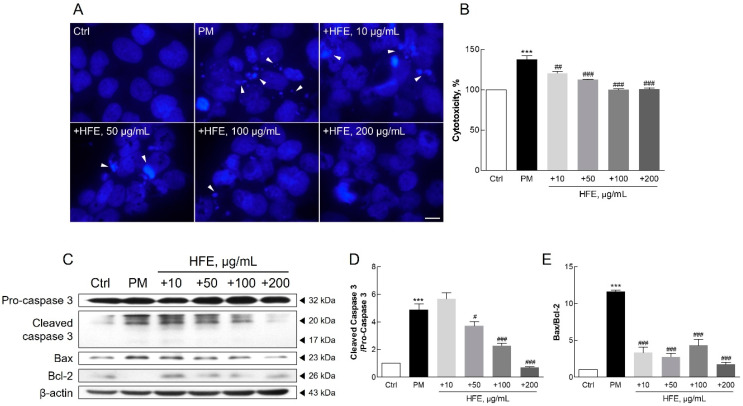
Effect of HFE on PM-induced apoptosis in HaCaT cells. Cells were pre-treated with HFE (1 h) and exposed to PM (50 µg/cm^2^, 24 h). (**A**) Nuclear morphology was visualized using Hoechst 33342 staining. Arrows: apoptotic nuclear condensation and fragmentation. Scale bar = 10 µm. (**B**) Cell cytotoxicity was measured using LDH release. (**C**–**E**) Expression of apoptosis-related proteins (Bcl-2, Bax, and cleaved caspase-3) was analyzed by Western blot. Data represent the mean ± SD (*n* = 3). *** *p* < 0.001 vs. control; # *p* < 0.05, ## *p* < 0.01, ### *p* < 0.001 vs. PM group. HFE, *H. fusiformis* ethanol extract; PM, particulate matter; LDH, lactate dehydrogenase.

Hoechst staining revealed that nuclear condensation and granulation, which are the hallmarks of apoptosis, were prominent in PM-treated cells. In contrast, cells pre-treated with HFE showed significantly reduced apoptosis (Figure 4A). This protective effect of HFE was further supported by cytotoxicity assays, in which reduced LDH release was observed in HFE-treated cells compared with that in cells exposed only to PM, indicating reduced cell death (Figure 4B). Western blot analysis of apoptosis-related proteins, including Bcl-2, Bax, and caspase-3, confirmed that HFE modulates apoptotic pathways. Specifically, PM treatment upregulated the expression of the pro-apoptotic markers Bax and cleaved caspase-3, whereas HFE pre-treatment upregulated the anti-apoptotic protein Bcl-2 and reduced cleaved caspase-3 activation (Figure 4C–E).

Previous studies have demonstrated that PM-induced skin damage involves multiple biological processes, including DNA damage, protein carbonylation, and lipid peroxidation. These effects are ultimately attributable to ROS generation and oxidative stress [10,41]. Additionally, exposure to PM causes mitochondrial swelling, endoplasmic reticulum stress, apoptosis, and autophagy in mouse skin tissues and HaCaT cells [10]. Increased ROS and inflammatory cytokine levels resulting from air pollution can trigger apoptosis in keratinocytes through matrix metalloproteinase (MMP) activation [40]. Furthermore, PM induces pro-inflammatory effects by generating ROS, leading to oxidative stress. Oxidative stress can disrupt mitochondrial function, resulting in electron leakage, further ROS production, mitochondrial membrane disruption, mitochondrial DNA damage, and impaired ATP synthesis [42]. Such mitochondrial disturbances contribute to shifts in apoptotic regulators, increasing proapoptotic factors, such as Bax, and activating the caspase cascade, ultimately leading to cell apoptosis [42,43,44].

Our findings support those of previous studies indicating that HFE exerts anti-apoptotic effects on keratinocytes by modulating oxidative stress and inflammation. These results suggested that *H. fusiformis* exerts protective effects through multiple mechanisms, including suppression of ROS generation, maintenance of mitochondrial function, and restoration of the balance between pro-apoptotic and anti-apoptotic signals disrupted by PM exposure.

### 2.5. Fatty Acids and Fucoidan Content in HFE

**Figure 5:**
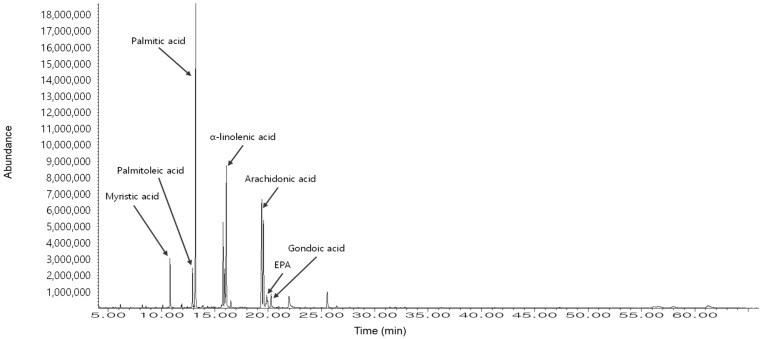
GC-MS analysis of the fatty acid composition in HFE. The chromatogram shows seven identified fatty acids (RT = 12.3–19.5 min): myristic acid (14:0), palmitoleic acid (16:1), palmitic acid (16:0), α-linolenic acid (18:3), arachidonic acid (20:4), EPA (20:5), and gondoic acid (20:1). The relative area percentage of each fatty acid was quantified using GC-MS (Table 1). The major components were palmitic acid (31.19%), α-linolenic acid (26.68%), and EPA (12.94%), which contribute to the antioxidant, anti-inflammatory, and anti-apoptotic properties of HFE against PM-induced skin damage.

Table 1 and Figure 5 show the GC-MS analysis results of the *H. fusiformis* extract, identifying seven major fatty acids, including palmitic acid and α-linolenic acid, which contribute to its antioxidant activity by scavenging ROS and enhancing antioxidant enzyme levels [10,41]. In particular, arachidonic acid and eicosatetraenoic acid (EPA) regulate inflammatory responses via the NF-κB and MAPK pathways [10,41], thereby reducing pro-inflammatory cytokine production. Furthermore, palmitic acid and α-linolenic acid stabilize cell membranes, while EPA inhibits apoptotic pathways [10,45], ultimately improving cell viability and reducing apoptosis [46]. These findings underscore the synergistic effects of fucoidan and fatty acids in mitigating PM-induced skin damage through antioxidant, anti-inflammatory, and anti-apoptotic mechanisms.

In addition, fucoidan in HFE was detected and quantified using a modified toluidine blue assay, yielding 1.2 ± 0.4% of the dried weight of *H. fusiformis*. This bioactive component has been well-documented for its prominent biological activities in seaweed extracts, particularly in reducing intracellular ROS levels and inflammatory markers in keratinocytes [46]. Furthermore, fucoidan-containing extracts demonstrate protective effects against PM-induced toxicity and apoptosis, notably by modulating cell-cycle progression in the sub-G1 phase [47]. The presence of fucoidan in HFE at this concentration is particularly pertinent because of its potent antioxidant and anti-inflammatory properties, which contribute substantially to the efficacy of the extract in protecting against PM-induced cellular damage [9].

## 3. Materials and Methods

### 3.1. Particulate Matter Preparation

National Institute of Standards and Technology (NIST) standard reference material 1649b (NIST1649b) was obtained from Sigma-Aldrich (St. Louis, MO, USA). To prepare the PM suspensions, the suspension was first sonicated in sterile phosphate-buffered saline (PBS) for 10 min to ensure even dispersion. A stock solution of PM (100 mg/mL) was prepared in PBS and subsequently diluted with the cell culture medium to the desired working concentrations for the experiments.

### 3.2. Sample Preparation and Content Analysis

Fresh *H. fusiformis* was collected from the eastern coast of the Republic of Korea. The collected specimens were authenticated by Professor Joo at Gangneung-Wonju National University, Gangwon, Republic of Korea. A voucher specimen was deposited in the herbarium of the College of Natural Science, Kangwon National University (KWNU80942).

Desalted and dried *H. fusiformis* was extracted with 75% ethanol at room temperature for 24 h with constant stirring. After extraction, the mixture was filtered through a Hyundai Micro No. 20 filter paper (Hyundai Micro, Seoul, Republic of Korea). The filtrate was concentrated under reduced pressure at 40 °C using a rotary evaporator. The concentrated extract was freeze-dried and stored at −20 °C for subsequent analyses. The final product, termed *H. fusiformis* ethanol extract (HFE), was reconstituted in dimethyl sulfoxide (DMSO) to a stock concentration of 100 mg/mL for use in experimental assays.

The fucoidan content in HFE was determined using a modified cationic dye method specifically designed to measure sulfated polysaccharides, as previously described [45,48]. For the assay, 10 µL of a fucoidan standard (Sigma-Aldrich, F8315; ranging from 0.1 to 1.5 mg/mL) or HFE diluted to 10 mg/mL was used. Each sample was mixed with 990 µL of toluidine blue solution (0.06 mM toluidine blue dye in 0.02 M maleic acid, adjusted to pH 1). The mixture was agitated vigorously to ensure thorough mixing. The absorbance of the resulting solution was measured at 632 nm using a microplate reader (Molecular Devices, San Jose, CA, USA). Fucoidan content was quantified and expressed in milligrams per 100 mg of HFE (%).

GC-MS analysis was performed using an Agilent 7890N GC with a 5975 MSD detector to evaluate the fatty acids. In brief, a HP-5MS column (30 m × 0.25 mm × 0.25 μm) was used with helium as the carrier gas (1 mL/min). The injection volume was 2 μL with a 2:1 split at 230 °C. The oven temperature was programmed from 100 °C to 250 °C. The ionization voltage was 70 eV with a mass range of 33–650 *m*/*z*. For fatty acid analysis, the extract was dissolved in distilled water and mixed with n-hexane (1:1 ratio), shaken vigorously, and left to separate. The upper n-hexane layer was collected, concentrated using a rotary evaporator, and subjected to methylation using BF₃-methanol complex (10–14%) at 60–70 °C for 10–15 min. The resulting fatty acid methyl esters were extracted with hexane, separated using saturated NaCl solution, and concentrated under nitrogen gas.

### 3.3. Antioxidant Assay

The antioxidant potential of HFE was assessed using the 2,2-diphenyl-1-picrylhydrazyl (DPPH) radical scavenging assay, a method renowned for its simplicity and quick evaluation of radical scavenging capacity. Samples were exposed to a 0.3 mM solution of DPPH (Sigma-Aldrich) at room temperature for 10 min. Absorbance was measured at 517 nm using a microplate reader. 100 μM ascorbate was used as positive control. The results were expressed as the percentage of radicals compared to the control.

To evaluate the protective effects of the proteins against hydroxyl radicals (OH^−^), a metal-catalyzed oxidation system was used. Hydroxyl radicals were generated by mixing 0.5 mM CuSO_4_ and 5 mM H_2_O_2_ at room temperature for 30 min. The radicals were then incubated with varying concentrations of the sample at 37 °C for 2 h. A standard protein, bovine serum albumin (BSA), was added to achieve a final concentration of 0.3 mg/mL, and the mixture was incubated at 37 °C for an additional 2 h. Ascorbate, at a concentration of 100 µM, was used as the control antioxidant. Following incubation, the reaction mixtures were analyzed using 10% sodium dodecyl sulfate–polyacrylamide gel electrophoresis (SDS-PAGE). The gels were stained with 0.1% Coomassie blue, and the intensity of the BSA protein bands was quantified using ImageJ software (Version 1.51j8; NIH, Bethesda, MD, USA). The intensity of the bands was related to the protective effects against oxidative damage, and the results are expressed as a percentage of the control group.

### 3.4. Cell Culture

HaCaT keratinocytes, a human keratinocyte cell line, were obtained from Dr. WK Kim at the Korea Institute of Science and Technology (KIST). The cells were maintained in Dulbecco’s modified Eagle’s medium (DMEM) supplemented with 10% fetal bovine serum (FBS) and antibiotics (100 U/mL penicillin and 100 µg/mL streptomycin), all sourced from Corning, NY, USA. The cells were cultured at 37 °C under a humidified atmosphere of 5% CO_2_. The medium was changed every 2–3 days based on the pH, color of the medium, and cell density to maintain optimal growth conditions. Consistency in cell culture conditions was meticulously monitored to ensure that the cells did not exceed 90% confluence. To ensure genetic stability and consistency in experimental outcomes, cells were used for experiments within the first 20 passages.

### 3.5. Cell Viability Assay

Cell viability was assessed using the Ez-Cytox Enhanced Cell Viability Assay Kit (WST-8; DoGenBio, Seoul, Republic of Korea). Cells were seeded at a density of 3.0 × 10^4^ cells/well in 96-well plates in serum-free DMEM. The cells were treated with various concentrations of the sample. After 24 h of treatment, the WST-8 assay reagent was added to each well. The plates were then incubated for an additional 2 h at 37 °C. The color intensity, which is indicative of cell viability, was quantified by measuring the absorbance at 450 nm using a microplate reader. The viability results were expressed as a percentage relative to the control group, which was set at 100% viability.

### 3.6. Cytotoxicity Assay

Cytotoxicity was determined using the CytoTox 96^®^ Non-Radioactive Cytotoxicity Assay Kit (Promega, Madison, WI, USA), which quantifies the release of lactate dehydrogenase (LDH) from lysed cells into the culture medium. For the assay, 50 µL of cell culture supernatant was transferred from each well to a new 96-well plate. An equal volume of the LDH reaction mixture was added to each well. The plates were incubated for 30 min at room temperature in the dark. The reaction was stopped by the addition of a stop solution, and the absorbance was measured at 490 nm using a microplate reader.

### 3.7. Nuclear Staining

Cells were seeded at a density of 5.0 × 10^4^ cells/well onto 8-well chamber slides (SPL Life Sciences, Gyeonggi, Republic of Korea) and allowed to stabilize for 24 h. After stabilization, the cells were treated with varying concentrations of the sample (10–200 µg/mL) for 2 h, followed by exposure to 50 µg/cm^2^ PM for 24 h. For nuclear staining, cells were first fixed using 4% paraformaldehyde for 15 min at room temperature. After fixation, the cells were permeabilized with 0.1% Triton X-100 in PBS for 10 min. Subsequently, the cells were stained with Hoechst 33342 (NucBlue™ Live ReadyProbes Reagent, Thermo Fisher Scientific, Waltham, MA, USA), as per the manufacturer’s instructions. The stained cells were visualized under an inverted fluorescence microscope (Eclipse Ti-S; Nikon, Tokyo, Japan) at ×600.

### 3.8. ROS Assay

Cellular oxidative stress was evaluated using 2′,7′-dichlorodihydrofluorescin diacetate (DCFH_2_-DA; Sigma-Aldrich, D6883). DCFH_2_-DA is a cell-permeable probe that is converted into highly fluorescent DCF upon oxidation, providing a measure of intracellular ROS levels. Cells were seeded in 96-well black plates with clear bottoms (SPL Life Sciences) at a density of 3.0 × 10^4^ cells/well and cultured under standard conditions (37 °C, 5% CO_2_) for 24 h to allow for stabilization. After stabilization, the cells were pre-treated with HFE at concentrations ranging from 10 to 200 µg/mL for 1 h. After treatment, the cells were incubated with 20 μM DCFH_2_-DA for 30 min, followed by PM at a concentration of 50 µg/cm^2^ to induce ROS production. Fluorescence intensity was measured using a Victor X2 fluorescence reader (PerkinElmer, Waltham, MA, USA) at excitation and emission wavelengths of 485 and 535 nm, respectively. Additionally, a qualitative assessment of ROS was performed by visualizing cellular fluorescence using an inverted fluorescence microscope at ×200.

### 3.9. qPCR Assay

Changes in gene expression were analyzed using quantitative polymerase chain reaction (qPCR). Total RNA was extracted from the cells using the TRIzol reagent (Thermo Fisher Scientific), according to the manufacturer’s guidelines. For cDNA synthesis, 1.0 µg of total RNA was utilized as a template using the ImProm-II Reverse Transcriptase System (Promega), following the prescribed protocol. qPCR was performed using SensiMix SYBR Hi-ROX PCR Master Mix (Bioline, London, UK), as previously described [49]. The relative expression levels of the target genes were quantified using the 2^−ΔΔCt^ method, with β-actin serving as the internal control to normalize the data. The results are presented as fold-changes relative to the control group. Primer sequences are listed in Table 2.

### 3.10. Western Blot Analysis

The cells were lysed in radioimmunoprecipitation assay (RIPA) buffer supplemented with protease and phosphatase inhibitors (Roche, Basel, Switzerland). Western blotting was performed as described previously [49]. Briefly, cell lysates (50 μg) were separated with 10% or 15% SDS-PAGE and transferred onto a polyvinylidene difluoride (PVDF) membrane. The membrane was blocked using 3% BSA in Tris-buffered saline with 0.1% Tween-20 solution (TBS-T) and incubated with specific primary antibodies (4 °C, overnight), followed by incubation with horseradish peroxidase (HRP)-conjugated secondary antibodies (room temperature, 2 h). Protein bands were visualized using an enhanced chemiluminescence (ECL) solution (Thermo Fisher Scientific), and luminescent signals were captured and quantitatively analyzed using ImageJ software. The results are expressed as relative protein expression and presented as fold-change compared to the control group. The antibodies used are listed in Table 3.

### 3.11. Statistical Analysis

All data are presented as means ± standard deviation (SD) of at least three independent experiments. Statistical significance between experimental groups was analyzed using one-way analysis of variance (ANOVA), followed by Tukey’s post hoc test using GraphPad Prism Version 5.01 (GraphPad Software Inc., San Diego, CA, USA). Differences were considered statistically significant at *p* < 0.05.

## 4. Conclusions

In the present study, we investigated the protective effects of HFE against PM-induced damage in human keratinocytes. Our findings demonstrate that HFE exhibits crucial antioxidant and ROS scavenging properties while modulating key inflammatory and apoptotic pathways. This aligns with existing literature highlighting the protective properties of *H. fusiformis* and other seaweed extracts against environmental pollutants.

The inclusion of fatty acid analysis provided additional mechanistic insights, highlighting the role of key fatty acids such as α-linolenic acid, EPA, and palmitic acid. These fatty acids contributed to the extract’s ability to mitigate oxidative stress, modulate inflammatory signaling, and protect cellular integrity. The synergistic action of fucoidan and these fatty acids enhanced the overall efficacy of HFE, suggesting its potential as a natural anti-pollution skincare agent by modulating inflammatory and apoptotic pathways. Future studies focusing on in vivo validation and formulation optimization are necessary to realize its dermatological potential.

Among the bioactive compounds identified, the seven fatty acids, particularly palmitic acid and oleic acid, have been reported to modulate oxidative stress pathways by directly scavenging reactive oxygen species and enhancing endogenous antioxidant defenses. Likewise, fucoidan demonstrated significant anti-inflammatory effects by inhibiting MAPK activation and downregulating inflammatory gene expression, which aligns with our observed results. Although our study primarily assesses the overall efficacy of HFE, these findings suggest that the identified compounds may play pivotal roles in the observed protective effects. Further mechanistic studies are warranted to precisely delineate their individual contributions to HFE’s therapeutic potential.

In addition to fatty acids, fucoidan—a major bioactive component of *H. fusiformis*—further enhances HFE’s protective effects by modulating inflammatory and apoptotic pathways. Our results suggest that HFE restores the balance between pro- and anti-apoptotic signals disrupted by PM exposure by virtue of its antioxidant and anti-inflammatory properties. These findings highlight the potential of HFE, specifically its fucoidan component, as a natural protective agent against PM-induced skin damage.

However, further in vivo studies and mechanistic investigations are warranted to validate these findings and explore the potential therapeutic applications of HFE in mitigating the adverse effects of PM on human skin. Future studies should also focus on optimizing the HFE dosage, evaluating long-term effects, and comparing its efficacy with existing treatments. Additionally, exploring different formulations and delivery methods of HFE may further enhance its practical applicability in dermatological settings.

**Figure 6:**
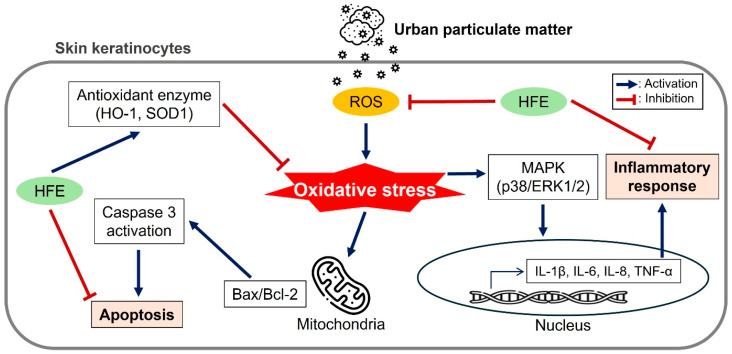
Schematic summary of the protective mechanisms of HFE against PM-induced damage in HaCaT keratinocytes. The diagram illustrates three major protective pathways: (1) antioxidant effect via ROS reduction and antioxidant enzyme upregulation, (2) anti-inflammatory effect through MAPK pathway inhibition, and (3) anti-apoptotic effect via caspase suppression. HFE, *H. fusiformis* ethanol extract; PM, particulate matter; ROS, reactive oxygen species; MAPK, mitogen-activated protein kinase.

In conclusion, this study demonstrates that HFE effectively protects human keratinocytes against PM-induced damage through its antioxidant, anti-inflammatory, and anti-apoptotic activities as illustrated in Figure 6. Although current limitations necessitate further research, the demonstrated efficacy of this marine-derived natural compound indicates significant potential for anti-pollution skincare applications. The development of HFE-based products may provide an environmentally sustainable solution for protecting skin health in urban populations exposed to elevated levels of air pollution. Furthermore, these findings suggest that purified HFE holds promise as a potential cosmeceutical ingredient for anti-pollution skincare formulations, given its multifunctional protective effects against PM-induced oxidative stress, inflammation, and apoptosis. This research not only advances our understanding of natural photoprotective compounds but also lays the groundwork for sustainable cosmeceutical innovations in urban skincare.

## Figures and Tables

**Table 1:** GC-MS analysis of fatty acid composition in HFE.

No.	Composition	Common Name	Library Name	Relative Area%
1	14:0	Myristic acid	Tetradecanoic acid, methyl ester	4.42
2	16:1	Palmitoleic acid	9-Hexadecenoic acid, methyl ester	4.11
3	16:0	Palmitic acid	Hexadecanoic acid, methyl ester	31.19
4	18:3	α-Linolenic acid	9,12,15-Octadecatrienoic acid, methyl ester	26.68
5	20:4	Arachidonic acid	5,8,11,14-Eicasatetraenoic acid, methyl ester	18.03
6	20:5	EPA	5,8,11,14,17-Eicosapentaenoic acid, methyl ester	12.94
7	20:1	Gondoic acid	11-Eicosenoic acid, methyl ester	2.64

**Table 2:** Primer sequences for qPCR assays.

Species	Gene	Primer Sequences		Accession No.
Human	HO-1	Fwd	5′-CTT CAC CTT CCC CAA CAT TG	NM_002133.3
		Rvs	5′-CCT CAA AGA GCT GGA TGT TG	
	SOD1	Fwd	5′-GCA TCA TCA ATT TCG AGC AGA	NM_000454.5
		Rvs	5′-CAA TAG ACA CAT CGG CCA CAC	
	IL-1β	Fwd	5′-GTA CCT GAG CTC GCC AGT GA	NM_000576.3
		Rvs	5′-TGA AGC CCT TGC TGT AGT GG	
	IL-6	Fwd	5′-CCT AGA GTA CCT CCA GAA CA	NM_001318095.2
		Rvs	5′-AGA TGA GTT GTC ATG TCC TG	
	IL-8	Fwd	5′-CAA ACC TTT CCA CCC CAA AT	NM_000584
		Rvs	5′-ACA ACC CTC TGC ACC CAG TT	
	TNF-α	Fwd	5′-TTG TTC CTC AGC CTC TTC TC	NM_000594.4
		Rvs	5′-AAG ATG ATC TGA CTG CCT GG	
	β-Actin	Fwd	5′-ACC TGA CTG ACT ACC TCA TG	NM_001101.5
		Rvs	5′-CTC ATT GCC AAT GGT GAT GA	

**Table 3:** Antibody list for western blot analysis.

Epitope	Manufacturer	Cat. No.	Dilution	Host
β-Actin (43 kDa)	Santa Cruz Biotechnology	sc-81178	1:1000	Mouse
Bax (23 kDa)		sc-70407	1:1000	Mouse
Bcl-2 (26 kDa)		sc-7382	1:1000	Mouse
Caspase-3 (32/20/17 kDa)		sc-56053	1:1000	Mouse
p-p38 (38 kDa)		sc-7973	1:1000	Mouse
p38 (38 kDa)		sc-535	1:1000	Rabbit
p-ERK1/2 (44/42 kDa)	ABclonal	AP0974	1:1000	Rabbit
EKR1/2 (44/42 kDa)		A16686	1:1000	Rabbit
Mouse IgG (HRP-linked)	Cell Signaling Technology	#7076	1:5000	Goat
Rabbit IgG (HRP-linked)		#7074	1:10000	Horse

## Data Availability

Data are contained within the article.

## Abbreviations

ANOVA	Analysis of variance
ARE	Antioxidant response element
BSA	Bovine serum albumin
DCFH_2_-DA	2′,7′-Dichlorodihydrofluorescin diacetate
DMEM	Dulbecco’s modified Eagle’s medium
DMSO	Dimethyl sulfoxide
DPPH	2,2-Diphenyl-1-picrylhydrazyl
ECL	Enhanced chemiluminescence
ERK	Extracellular signal-related kinase
FBS	Fetal bovine serum
HFE	*Hizikia fusiformis* ethanol extract
HO-1	Heme oxygenase 1
HRP	Horseradish peroxidase
IL	Interleukin
LDH	Lactate dehydrogenase
MAPK	Mitogen-activated protein kinase
MMP	Matrix metalloproteinase
NF-κB	Nuclear factor κ-light-chain-enhancer of activated B cells
NRF2	Nuclear factor erythroid 2-related factor 2
PBS	Phosphate-buffered saline
PM	Particulate matter
PVDF	Polyvinylidene difluoride
qPCR	Quantitative polymerase chain reaction
SD	Standard deviation
SDS-PAGE	Sodium dodecyl sulfate–polyacrylamide gel electrophoresis
SOD1	Superoxide dismutase 1
TLR	Toll-like receptor
TNF α	Tumor necrosis factor α
